# Supplementary material for: Identification of photoperiod-induced specific miRNAs in the adrenal glands of Sunite sheep (Ovis aries)
Source: Front Vet Sci. 2022 Jul 22;9:888207. doi: 10.3389/fvets.2022.888207 (PMC9354845; doi:10.3389/fvets.2022.888207)
Supplement: Supplementary file 1 [file Data_Sheet_1.ZIP › Supplementary file/miRDeep2 file.zip!/miRDeep2 file/Know_Structure/SPLP42A1/oar-mir-21.pdf]

miRBase precursor : oar-mir-21
Total read count : 189100
oar-miR-21 read count : 189088
remaining reads : 12

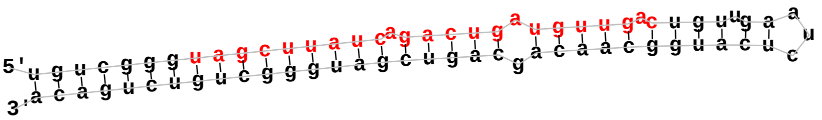

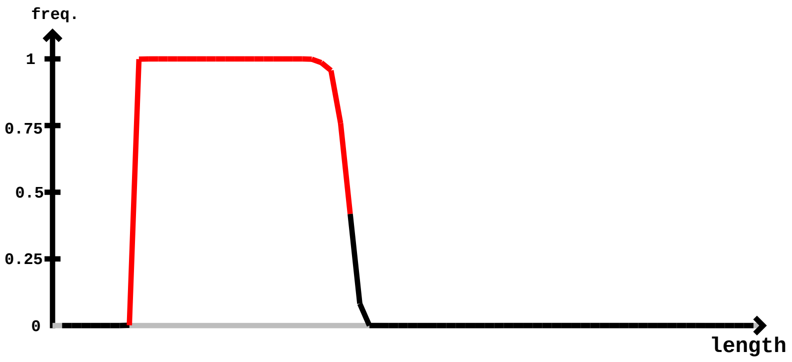

oar-miR-21

5'- ugucggguagcuuaucagacugauguugacuguugaaucucauggcaacagcagucgaugggcugucugaca -3'	exp		
((((((((((((((((.(((((.(((((.((((.((...))))))))))).)))))))))))))))))))))	reads	mm	sample
......guagcuuaucagacugaug...	1	0	seq
......guagcuuaucagacugaugu..	5	0	seq
......guagcuuaucagacugauguu...	6	0	seq
......guagcuuaucagacugauguug..	97	0	seq
......guagcuuaucagacugauguuga...	70	0	seq
......guagcuuaucagacugauguugac..	17	0	seq
......guagcuuaucagacugauguugacu.......................................	8	0	seq
......guagcuuaucagacugauguugacuguu....................................	1	0	seq
.......uagcuuaucagacugau..	11	0	seq
.......uagAuuaucagacugau..	1	1	seq
.......uagcuuaucagacugCu..	1	1	seq
.......uagcuuaucagacugaug...	201	0	seq
.......uagcuuaucagacugaugu..	2460	0	seq
.......uagcuuaucagacugauguu...	5531	0	seq
.......uagcuuaucagacugauguug..	37237	0	seq
.......uagcuuaucagacugauguuga...	64241	0	seq
.......uagcuuaucagacugauguugac..	63535	0	seq
.......uagcuuaucagacugauguugacu.......................................	15467	0	seq
.......uagcuuaucagacugauguugacug......................................	11	0	seq
.......uagcuuaucagacugauguugacugu.....................................	6	0	seq
.......uagcuuaucagacugauguugacuguu....................................	3	0	seq
.......uagcuuaucagacugauguugacuguug...................................	1	0	seq
.......uagcuuaucagacugauguugacuguugaau................................	1	0	seq
........agcuuaucagacugauguu...	3	0	seq
........agcuuaucagacugauguug..	34	0	seq
........agcuuaucagacugauguuga...	53	0	seq
........agcuuaucagacugauguugac..	46	0	seq
........agcuuaucagacugauguugacu.......................................	17	0	seq
........agcuuaucagacugauguugacuguug...................................	1	0	seq
.........gcuuaucagacugauguu...	1	0	seq
.........gcuuaucagacugauguug..	9	0	seq
.........gcuuaucagacugauguuga...	6	0	seq
.........gcuuaucagacugauguugac..	3	0	seq
.........gcuuaucagacugauguugacu.......................................	1	0	seq
..........cuuaucagacugauguuga...	1	0	seq

oar-miR-21

```
ugucggguagcuuaucagacugauguugacuguugaaucucauggcaacagcagucgaugggcugucugaca
...........uuaucagacugauguugac..........................................    1           0           seq
...........uuaucagacugauguugaU..........................................    1           1           seq
.............................................caacagcagucgaugggc.........    1           0           seq
.............................................caacagcagucgaugggcugu......    6           0           seq
.............................................caacagcagucgaugggcuguc.....    3           0           seq
.............................................caacagcagucgaugggcugucu....    1           0           seq
```
